# Clean Milk or Pasteurised Milk

**Published:** 1908-03-07

**Authors:** 


### The Hospital

A JOURNAL OF

The Medical Sciences and Hospital Administration.

NEW SERIES. No. 51, VOL. II. [No. 1123, VOL. XLIII.] SATURDAY, MARCH 7, 1908.

### CLEAN MILK OR PASTEURISED MILK?

Of twenty-four samples taken at Liverpool Street in November last, thirteen were fairly clean, nine were dirty and unsatisfactory, and two were tuberculous. —Report of the Medical Officer of Health for the City, published February 14, 1908.

The representative of Mr. Nathan Straus is paying a visit to this country, and is making inquiries with a view to establishing depôts for the supply of pasteurised milk to the poor in London and Ireland.

IN the above paragraphs the crux of the milk supply is sharply outlined. Much ink has been spilt on the iniquities of milk dealers, and at length there appears to be hope that a serious attempt will be made to deal with the problem, and that in the happy future we shall neither be condemned to feed our infant population on a dirty, septic, drug-contaminated fluid, nor have to continue our efforts to render such a fluid as harmless as possible. The crying need of the infant for *clean* milk is realised by all who have to deal with infantile ailments. Yet even our children's hospitals are by no means perfect in the steps they take to ensure such a supply. Recently the committee, appointed by the Central Hospital Council for London, has recommended that the hospitals should adopt a form of contract with the milk dealers which will ensure a supply of pure, clean milk from healthy cows, unadulterated, unpasteurised, cooled down to at least 60° F. after milking, and containing 3.25 to 3.50 per cent. of fat. This may be taken as an authoritative expression of opinion of the necessity for pure, clean milk which is not manipulated in any way by the addition of preservatives to prevent the growth of micro-organisms or by heat to destroy those already present. On the other hand, Mr. Straus proposes to start depôts for the supply of pasteurised milk, as he has already done in America and Germany. He has received a considerable amount of encouragement from the press and from distinguished individuals who are always ready to support philanthropic schemes on sentimental grounds, without weighing the value of the steps suggested or taking the opinion of experts on the subject.

The history of the milk supply since the development of bacteriology is an interesting one. At first it was deemed essential to destroy all the microbes present by prolonged sterilisation. Later on, this high temperature was realised to be a disadvantage in that it devitalised milk and made it less nutritious. Now there are two schools of thought. Some hold that milk should be given fresh. Others maintain that, because of the impurities so often present, it should invariably be heated to a high temperature, a few being satisfied with boiling, in order to destroy the germs of tuberculosis, typhoid fever, etc. Eventually what we hold to be the rational view will prevail, that the milk must be pure, clean, unadulterated, and unpreserved by heat. Mr. Straus's views on the value of pasteurised milk are those held by several members of the profession many years ago, and still held by a few who have not advanced since those days. Just as the dependence of the specialist in infant feeding on a rigid percentage system of laboratory and American development, and on pasteurisation, was in the past thought imperative, so there are still physicians who rely on these ancient methods although they have been largely dropped in the country of their origin, and are not supported by the experts on children's diseases in this country. Commercial pasteurised milk is, in the opinion of many, much more dangerous than the ordinary milk supply. In the process of making it, the lactic acid bacilli are destroyed, as well as those organisms which may give rise to infective disease. But there is this unfortunate result. The lactic acid bacilli grow with great rapidity in milk and rapidly crowd out other organisms. The lactic acid turns the milk sour, a change appreciable to the smell and taste of the most ignorant and stupid. Sour milk does not go putrid for a long time. Moreover, there is much evidence in support of the value of lactic acid as an intestinal antiseptic, or preventive of the growth of injurious intestinal flora. If these bacilli are destroyed by heat, then the spores of other and more dangerous organisms will grow, and often there are no olfactory or gustatory signs of the deadly changes which result. The spores of the bacillus enteritidis sporogenes are only destroyed by very prolonged heating to 100° C., and the presence of this organism is a serious source of diarrhœa. Further, the pasteurisation of milk enables much that is stale and deleterious to be sold as, or mixed with, fresh milk. We may sum up the disadvantages of pasteurised milk as increasing the liability to diarrhœal diseases, to scorbutic affections, anæmia, rickets, and malnutrition generally. Though we cannot approve of the direction of Mr. Straus's philanthropy in this direction, we might point out that there is a crying need for the establishment, in connection with every children's hospital, of a milk depôt for the supply of pure milk, which can be modified in accordance with the directions of the physician in charge of the case. The majority of infants brought to the out-patient departments require such a supply rather than drugs. Unfortunately the hospitals cannot afford the initial outlay necessary. Such a supply need not lead to pauperisation, for a charge to cover the actual cost could be made to those mothers who were in a position to pay, and the supply should only be gratuitous to the most necessitous.

